# A Publicist on Dr. Wiley

**Published:** 1911-09

**Authors:** 


### EDITORIAL DEPARTMENT.

THE EDITOR of the "Red Back" having been in the deep tangled wildwood of San Marcos in pursuit of health, strength and the elusive black bass—two months, and in that time not having seen a medical journal—feels that he is away behind as to happenings. He will catch on though, pretty quick, and you may expect hereafter to hear the "crack, crack" of his rapid-fire gun, all along the line, as of yore. The fact is, that on that trip he contracted a terrible case of lazy, for which he is now undergoing treatment. A young colt, like ye editor, allowed to run in clover and alfalfa pastures, in sweet fields arrayed in living green by rivers of delight, backs his ears and lifts threatening heels at the approach of the bridle or harness. But—he must come down to a steady gait by next time. Wait, wait till the tan and lazy wear off and he'll start something that will make your head swim.

---

A PUBLICIST ON DR. WILEY.—I publish herewith a lengthy letter from a Mr. Harris of New York, whose letterheads announce him as a "Publicist." A publicist is defined by Webster to be a writer on the laws of nature and of nations, one versed in the science of public right, the principles of government, etc. A pronouncement from such an eminently respectable person as one so versed, is entitled to great consideration. In fact, it would seem to be ex cathedra, and should settle the question of Dr. Wiley's fitness or unfitness. It may be that Mr. Harris, being versed in the science of public right and the principles of government, is adviser to Congress, the President, the Secretary of Agriculture and all other officials, and that he has pronounced Dr. Wiley unfit. I have no idea who Mr. Harris is nor what his affiliations are, nor do I know how publicist-ing is made to pay; but I am that rash, perhaps foolhardy, that, like the old preacher who begged to differ with Brother Paul, I must differ from the dictum of this eminent publicist. However, regardless of the merits or otherwise of Mr. Harris' arguments which, to me, seem strained to make a case against Dr. Wiley, and to echo the cry of those hit hard by Dr. Wiley's decisions, the TEXAS MEDICAL JOURNAL is open, always, to both parties to a controversy, and it is only fair to let the other fellow be heard. Mr. Harris lays stress on the fact, he says it is a fact, that Dr. Wiley is not a physician—"could not qualify as a physician." That seems to be the head and front of his offending, coupled with his unpardonable effrontery (?) in differing from the great chemists, Crittenton and Remsen. Need a chemist be a physician to be capable and expert? Has he no right to differ in opinion from the decision of those gentlemen—as much right as Judge Harlan had to differ from White's "funny,"—biased opinion, or as Publicist Harris, as to that matter, has to differ from him (Wiley) he, (Harris) being, as I take it, neither physician nor chemist. [In last issue I wrote,—speaking of this controversy—"No thief ever felt the halter draw and had a good opinion of the law." The printer got it "nothing" ever felt the halter draw, etc.]

---

The Passing of Simmons.—So the boss is down and out as Secretary of the American Medical Association; but he still runs the great *Octopus* and will develop a great publishing business. I hope we have heard the last of him, and that peace will dwell perpetually in the ranks. A Dr. Craig becomes Secretary.

---

What's the Matter With the A. M. A.?—*The Texas State Journal of Medicine* says: "There were in the neighborhood of 3000 physicians in attendance upon the Los Angeles meeting in June." My! "how she has shrunk." Time was when 6000—7000—attended—out of a membership of 30,000. After all the drumming, horn-blowing and pull of passenger agents—3000—or about 10 per cent were induced to go.

---

Married at Rockport, Texas, July 20, Dr. Jno. Wesley Crutcher to Owene Elizabeth Peeler, daughter of Mr. and Mrs. A. J. Peeler of Houston. "At home" at Mineral Wells, Texas, after August 5.

---

If a patient with the signs and symptoms of chronic appendicitis is unduly anemic and has lost weight, be prepared for the possibility of a more serious lesion, e. g., neoplasm, ileocecal tuberculosis.—*American Journal of Surgery.*

---

Toothache or Earache Remedy.—Take dram vial and fill with pure water; then drop a tablet of atropin, 1-100 gr., into it. When dissolved it is ready for use. Drop two or three drops into the tooth or ear, as the case may be, and plug with absorbent cotton. Repeat every thirty minutes till relieved.—*Medical Brief.*

